# 152 fs nanotube-mode-locked thulium-doped all-fiber laser

**DOI:** 10.1038/srep28885

**Published:** 2016-07-04

**Authors:** Jinzhang Wang, Xiaoyan Liang, Guohua Hu, Zhijian Zheng, Shenghua Lin, Deqin Ouyang, Xu Wu, Peiguang Yan, Shuangchen Ruan, Zhipei Sun, Tawfique Hasan

**Affiliations:** 1 Shenzhen Key Laboratory of Laser Engineering, College of Optoelectronic Engineering, Shenzhen University, Shenzhen 518060, China; 2 Cambridge Graphene Centre, University of Cambridge, Cambridge CB3 0FA, United Kingdom; 3 Department of Micro- and Nanosciences, Aalto University, Aalto, FI-00076, Finland

## Abstract

Ultrafast fiber lasers with broad bandwidth and short pulse duration have a variety of applications, such as ultrafast time-resolved spectroscopy and supercontinuum generation. We report a simple and compact all-fiber thulium-doped femtosecond laser mode-locked by carbon nanotubes. The oscillator operates in slightly normal cavity dispersion at 0.055 ps^2^, and delivers 152 fs pulses with 52.8 nm bandwidth and 0.19 nJ pulse energy. This is the shortest pulse duration and the widest spectral width demonstrated from Tm-doped all-fiber lasers based on 1 or 2 dimensional nanomaterials, underscoring their growing potential as versatile saturable absorber materials.

Ultrafast fiber lasers have been extensively studied and developed over the past decades^1^,^2^. Their continuous improvements have started to challenge other laser technologies due to their compact design, inherently high beam quality and alignment-free format^1^,^2^. In recent years, ultrafast lasers at 2 μm spectral region are attracting growing attention due to their various applications, including remote gas sensing, bio-medical treatment, mid-infrared (IR) supercontinuum generation and mid-IR frequency comb. Thulium- (Tm) and holmium- (Ho) doped gain fibers are known to have lasing spectrum in this spectral region. In particular, Tm-doped fibers offer a broad gain bandwidth ranging from 1.8 to 2.1 μm^3^, enabling ultrashort (*e. g.* < 200 fs) pulse generation from oscillators based on these fibers. Such ultrashort pulses are highly demanding for ultrafast time resolved spectroscopy, which can be used to monitor dynamic relaxation processes of materials^4^. In addition, highly efficient nonlinear mid-IR frequency conversion can be enabled with ultrashort pulses at 2 μm region due to their high peak power^5^.

It is widely accepted that cavity group delay dispersion (GDD) control is of critical importance for ultrashort pulse generation. In general, a fiber cavity with negative GDD can generate soliton pulses, due to the balance of negative dispersion and self-phase modulation. Soliton duration is typically determined by the cavity dispersion such that τ∝|GDD**|**^1/2^ 2. Pulses as short as 58 fs at 1.9 μm have been obtained by designing a short fiber cavity with only −0.017 ps^2^ GDD^6^. However, bulk optics is employed in this cavity, eliminating the advantage offered by the all-fiber configuration. To date, the shortest soliton duration reported from an all-fiber Tm-doped oscillator is 190 fs^7^, however, with a limited (20 pJ) pulse energy. An effective way to generate shorter pulses with moderate pulse energy is to utilize intracavity dispersion management that typically set total cavity GDD close to zero^8^ or slightly normal^9^,^10^. Combining dispersion management with nonlinear polarization evolution (NPE) technique, sub-200 fs pulses have been successfully achieved in Tm-doped^11^,^12^,^13^,^14^,^15^ and Ho-doped fiber lasers^16^. However, they all require the use of bulk optics to realize NPE. Dispersion management technology has also been employed in many reports on all-fiber, ultrashort Tm-doped oscillators^17^,^18^,^19^,^20^,^21^. For example, ref. [17] used a chirped Bragg fiber grating to manage dispersion, both soliton and dissipative soliton regimes can be achieved in a semiconductor saturable absorber mirror (SESAM)-based Tm oscillator. Ref. [19] combined SESAM and nonlinear amplifying loop mirror (NALM) mechanisms to mode-lock an all-fiber Tm laser and directly achieved 2 nJ pulses with 230 fs duration. Ref. [20] reported the highest single pulse energy (4.9 nJ) from an all-fiber laser cavity at 2 μm regime. Recently, Sobon *et al*. used the NPE technique to directly generate dissipative solitons with >100 nm bandwidth. This is thus far the broadest spectrum directly generated from a normal dispersion mode-locked Tm-doped fiber laser^21^. To date, the shortest pulse directly generated from an all-fiber Tm-doped oscillator is 140 fs, mode-locked by SESAM^18^.

Although NPE^3^ and SESAMs^7^ are the two most widely studied mechanisms for mode-locking Tm-doped fiber lasers, they still suffer from some fundamental drawbacks. For example, NPE requires polarization optimization at all times due to its environmental sensitivity. SESAMs generally have a limited bandwidth which results from their resonant design and require complex semiconductor fabrication processes, packaging and integration^22^. Recently, a new generation of saturable absorber (SA) based on 1D (carbon nanotubes, CNTs) or 2D (such as graphene, MoS_2_ and black phosphorous) nanomaterials have come into prominence^23^,^24^,^25^,^26^,^27^,^28^,^29^,^30^,^31^,^32^,^33^,^34^,^35^,^36^,^37^,^38^. These nanomaterial-based SAs have the advantages through easy and economic fabrication and when in composite form, transmission-type operation for simple integration into all-fiber ring cavities^33^,^39^. Among these nanomaterials, CNTs possess ultrafast recovery time, large modulation depth and wide operation bandwidth when samples with large diameter distribution are used^24^. Such diameter distribution are inherent in most CNT production techniques^40^. Thus, fiber lasers mode-locked by CNTs have been widely demonstrated in the 1 41,42,43, 1.5 23,44,45,46 and 2 μm^25^,^30^,^47^,^48^,^49^ region. However, because of the large negative dispersion of SMF fiber at 2 μm, CNT-mode-locked Tm-doped fiber oscillators typically emit picosecond pulses^25^,^29^,^47^. Recently, a section of germanium-silicate (GeO_2_/SiO_2_) fiber was used to control the intracavity dispersion, 450 fs pulses with 15.8 nm bandwidth were directly achieved in a CNTs/NALM hybrid mode-locked Tm-doped fiber laser^19^. This is thus far the shortest pulse duration with the broadest bandwidth directly generated from a Tm-doped fiber oscillator mode-locked by CNTs or other 2D nanomaterials.

In this letter, we demonstrate a robust and compact Tm-doped all-fiber femtosecond oscillator mode-locked by CNTs. The oscillator operates in a slightly normal GDD after intracavity dispersion management. 152 fs pulses with 52.8 nm bandwidth are directly produced from our oscillator without any free-space components. Both the pulse duration and spectral bandwidth are ~3 times shorter and wider, respectively, than previous CNT-based Tm-doped fiber lasers^19^, and demonstrate an improvement on graphene or other 2D material-based Tm-doped fiber oscillators^50^,^51^,^52^,^53^,^54^.

## Results

### CNTs preparation and characterization

**Figure 1:**
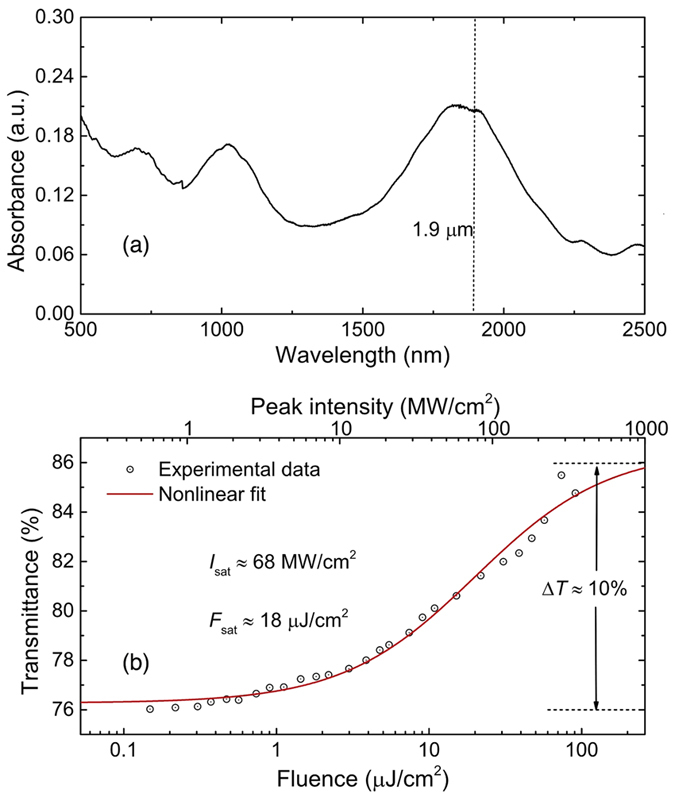
(**a**) Linear absorption spectrum and (**b**) nonlinear power-dependent absorption of CNT-SA.

We use CNTs grown by arc discharge on Ni/Y catalyst (P2, Carbon Solutions, Inc., 0.5–1.5 μm in length, 4–8% catalyst metal content)^55^. The CNTs, with diameters ranging from 1.3–1.6 nm, are embedded into carboxymethyl cellulose via solution processing to form polymer composite^40^. After 4 or 5 days of slow evaporation at room temperature, we obtain a free-standing polymer composite SA with ~30 μm thickness. Such composites can be used in transmission-type configurations through simple and easy integration into all-fiber format. These CNTs exhibit optical absorption in the 1.75 to 2 μm range centered at ~1.85 μm (see Fig. 1(a)) due to S_11_ excitonic absorption of the semiconducting tubes. This absorption band fits well with the operating wavelength of the Tm gain fiber (see dashed line in Fig. 1(a)). Power-dependent absorption at 1.9 μm of the CNT-SA are measured with an optical parametric oscillator (Coherent Chameleon Ultra II) producing ~260 fs pulses. The experimental data is then fitted by a nonlinear function of fast SA that describes its transmittance^9^, giving ~10% modulation depth and ~18 μJ/cm^2^ saturation fluence (corresponding to ~68 MW/cm^2^ saturation intensity); Fig. 1(b).

### Setup of mode-locked fiber laser

**Figure 2:**
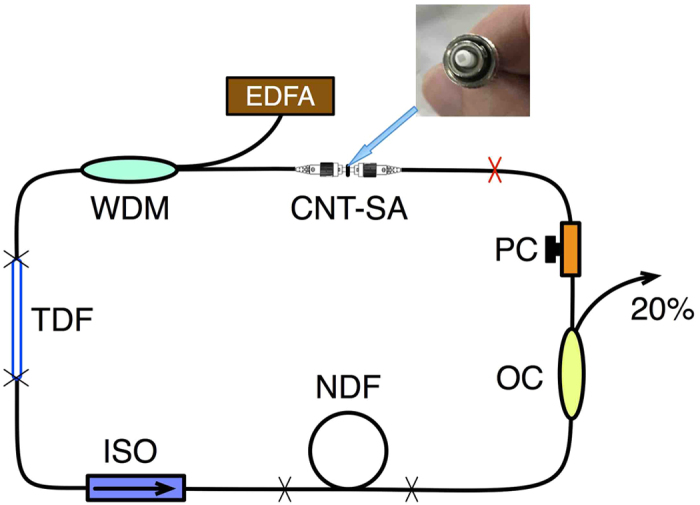
Laser setup. EDFA: erbium-doped fiber amplifier. WDM: wavelength division multiplexer. TDF: thulium-doped fiber. ISO: isolator. NDF: normal dispersion fiber. OC: optical coupler. PC: polarization controller. CNT-SA: carbon nanotube saturable absorber. Inset: photograph of an integrated CNT-SA.

The schematic of the laser setup is shown in Fig. 2. The CNT-SA is sandwiched between two fiber connectors (see inset of Fig. 2). The gain medium is a 17.5 cm long, highly-doped Tm fiber (Nufern SM-TSF-5/125) with −20 ps^2^/km dispersion at 1.9 μm estimated from numerical calculation. The pump is provided by a commercial Er fiber laser that emits at ~1570 nm and is coupled into the cavity via a 1570/1940 nm wavelength division multiplexer (WDM). The pigtails of the WDM amount to 1.14 m SMF-28e fiber. A polarization insensitive isolator, consisting of 0.91 m SMF-28e pigtail is spliced after the gain fiber and ensures unidirectional operation of the oscillator. An in-line polarization controller (PC) is employed to optimize the polarization state to compensate for the intrinsic weak birefringence of the passive and active fibers. 20% intracavity light is coupled out through a 20/80 coupler with 0.985 m long SMF-28e fiber in the cavity. The dispersion of the SMF-28e fiber is −71 ps^2^/km at 1.9 μm^15^.

Since the gain fiber and SMF exhibit negative dispersion at 1.9 μm, a component with normal dispersion, is required to realize dispersion management Ultra-high NA fibers, such as normal dispersion fiber (NDF). have been demonstrated to have normal dispersion at long wavelength, due to their much stronger waveguide dispersion than material dispersion^15^,^48^,^56^,^57^. We thus add a 3.75 m long fiber (Nufern UHNA4) as the NDF. The dispersion of UHNA4 fiber is measured to be 93 ps^2^/km at 1.9 μm^48^. Thus, the cavity length depicted in Fig. 2 is 6.96 m with a total GDD of 0.13 ps^2^. By changing the passive fiber between PC and CNT-SA via adding SMF-28e or NDF fibers and without changing other fiber segments, the net cavity GDD could be either normal or anomalous, within a range of 0.385 ps^2^ to −0.265 ps^2^. The total cavity length is varied between 6.96 m and 12.5 m.

### Mode-locking operation and characteristics

**Figure 3:**
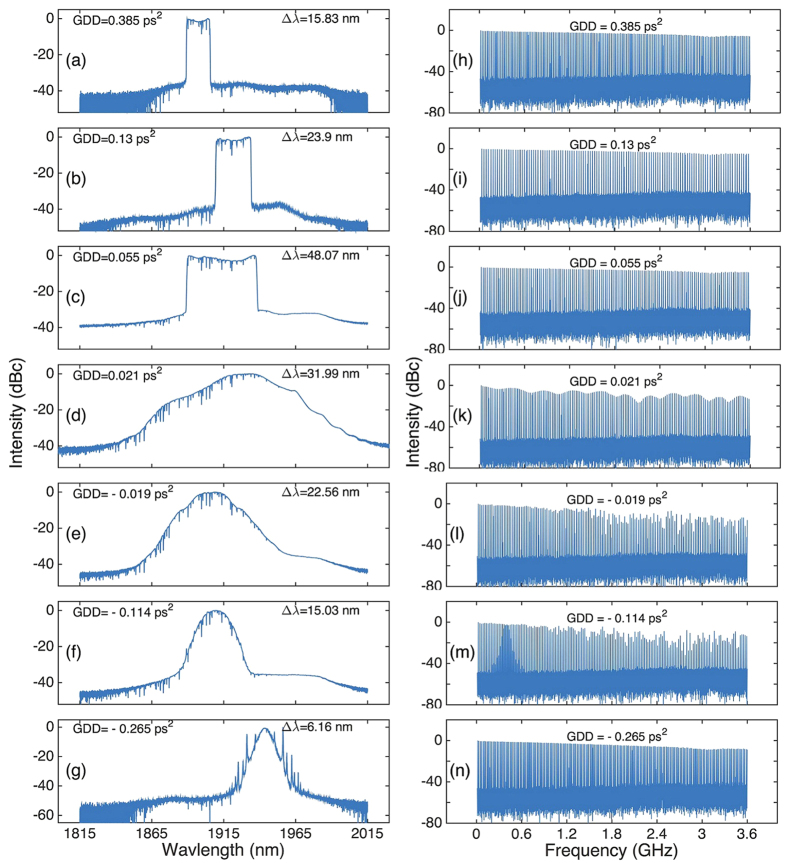
(**a–g**) Evolution of the output pulse spectrum and (**h–n**) wideband RF spectrum to up to 3.6 GHz (Resolution bandwidth: 100 kHz) with different cavity GDD.

When the pump power is increased to an appropriate level, stable oscilloscope traces could always be achieved by adjusting the PC in this dispersion range. We note that no pulse trace could be observed in the oscilloscope without the CNTs. This suggests that the CNT-SA plays a major role in starting and stabilizing the pulses. Figure 3(a–g) show the evolution of the pulse spectrum with different net cavity GDDs. Similar to previously reported 2 μm pulsed lasers^12^,^25^,^56^, sharp dips are observed due to the absorption of atmospheric molecules^58^. We experimentally find that the dips become significant when the wavelength is below 1940 nm, further confirming such dips are predominantly caused by the absorption of CO_2_ and water molecules^58^. Moreover, the central emission wavelength is usually blue-shifted from the peak emission wavelength of the gain fiber (1950~1970 nm) to ~1915 nm. This could be predominantly caused by the cavity loss. For example, the two splicing points between UHNA4 and SMF induce large insertion losses (~54% measured). Also, to get stable mode-locking operation, we sometimes bent a small segment of SMF-28e fiber to finely tune intra-cavity loss during the experiments. In addition, we note that the automatic balance between the intra-cavity dispersion and nonlinearity could play a role in the wavelength shift since it is sensitive to the wavelength and the setting of PC, respectively.

**Figure 4:**
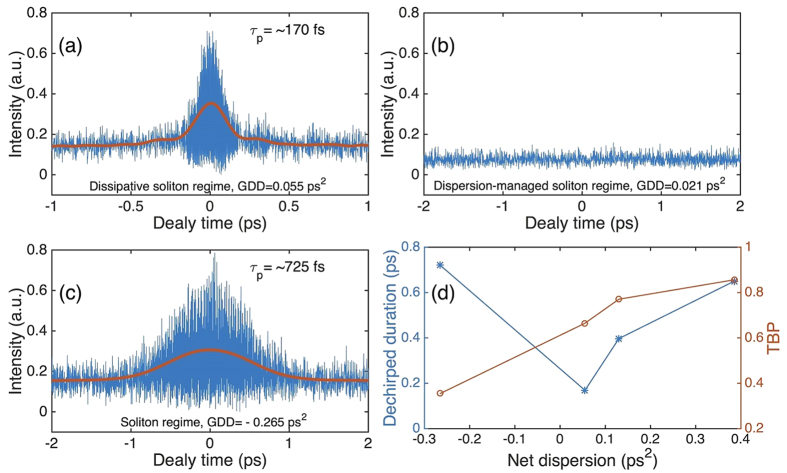
(**a–c**) AC/IAC traces of the dechirped pulse in three operating regimes. (IAC trace in blue, AC trace in red.). (**d**) The dechirped duration and TBP depend on the net dispersion.

Initially, a wide FWHM bandwidth of up to 48.07 nm is achieved with net cavity GDD of 0.055 ps^2^, accompanied by narrowing of the spectrum bandwidth on both sides of this dispersion value, see Fig. 3(a–g). On one hand, the steep spectral edges, expected as a signature of dissipative solitons^1^, are observed when the net GDD is larger than 0.055 ps^2^; Fig. 3(a–c). On the other hand, the absence of spectral sidebands in Fig. 3(d–f) show that these cavities work in the dispersion-managed soliton regime^2^. Finally, Kelly sidebands of soliton spectrum appears when the negative dispersion increases to −0.265 ps^2^; Fig. 3(g). We then use a radio-frequency (RF) spectrum analyzer (Agilent N9010) and a 12.5 GHz photodiode (EOT-5000) to verify the pulse stability of these cases by measuring their wideband RF spectra to up to 3.6 GHz; Fig. 3(h–n). RF spectra in high-order harmonics exhibit fluctuations in the dispersion-managed soliton regime (Fig. 3(k–m)), indicating a noise-like regime. This is confirmed by the autocorrelation (AC) trace/interferometric AC (IAC) trace measurements of three operating regimes (*i.e.* dissipative soliton regime, dispersion-managed soliton regime and soliton regime) since no stable pulse is generated in the dispersion-managed soliton regime, see Fig. 4(a–c). The noise-like operation could be caused by the limited modulation depth (~10%) of our CNT-SA^59^. Figure 4(d) summarizes the dechirped pulse duration and time-bandwidth product (TBP) as a function of net cavity dispersion. The shortest pulse duration of ~170 fs could be achieved as soon as the net dispersion is slightly normal. When the cavity works in the dissipative soliton regime, the TBP is >0.66 (*i.e.* >1.5 times of Fourier limit), indicating incomplete compression. Such imperfect compression could be caused by uncompensated high-order dispersion, in particular, the third-order dispersion induced by intra-cavity fibers (e.g., the NDF fiber)^15^.

**Figure 5:**
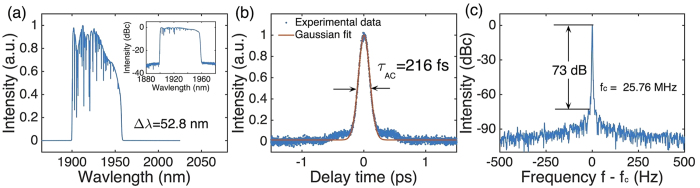
Experimental (**a**) output spectrum (Inset: optical spectrum on a logarithmic scale), (**b**) AC trace of the dechirped pulse, (**c**) RF spectrum at fundamental repetition rate (Resolution bandwidth: 1 Hz).

To further decrease the achievable pulse duration. We therefore increase the pump power and carefully optimize the polarization state of the cavity which can emit pulses with much wider bandwidth than other cavities (see Fig. 3(c)). The cavity has a length of 8.01 m, corresponding to 25.76 MHz pulse repetition rate. At 660 mW pump power, the oscillator delivers pulses with a 52.83 nm bandwidth, centered at 1927 nm, see Fig. 5(a). The output power is 4.85 mW, corresponding to 0.19 nJ pulse energy. A higher pump power results in multi-pulse operation or instability. This could be caused by over saturation of the CNT-SA. Normal net GDD of this oscillator is expected to produce positively chirped pulse^1^. Fig. 5(b) shows the AC trace of the pulse compressed with ~10.2 m long SMF-28e fiber. The AC FWHM is 216 fs. Assuming a Gaussian profile, the deconvoluted pulse width is ~152 fs, ~1.5 times of Fourier-limit. Considering the large bandwidth of 52.8 nm, we note that transform-limited <100 fs pulse is possible after compression, with appropriate high-order dispersion compensation (*e.g.*, prisms). Figure 5(c) shows the RF spectrum at the fundamental repetition rate. The SNR is as high as 73 dB, comparable to those previously reported pulsed lasers at 2 μm^12^,^15^,^48^, indicating stable mode-locking operation.

In summary, we have demonstrated a simple and compact all-fiber Tm-doped laser delivering 152 fs pulses with a 52.8 nm bandwidth. These are by far the shortest pulses with the broadest bandwidth, directly from CNT mode-locking Tm-doped fiber lasers, constituting a 3-fold improvement both in pulse duration and spectral bandwidth compared to previous reports. Such pulses have a great potential as low-cost light sources for mid-IR supercontinuum generation and pump-probe technique which typically requires ultrashort and wide bandwidth pulses. In addition, these pulses may also find applications as ideal seed pulse for chirped pulse amplification (CPA) because of its clean sidebands.

## Additional Information

**How to cite this article**: Wang, J. *et al*. 152 fs nanotube-mode-locked thulium-doped all-fiber laser. *Sci. Rep.* **6**, 28885; doi: 10.1038/srep28885 (2016).
